# Society Meetings

**Published:** 1904-04

**Authors:** 


### SOCIETY MEETINGS.

Dr. George L. Richards, of Fall River, Mass., ex-president of the Laryngo-otological section of the A. M. A., and author of Nose and Throat Work in General Medicine, lectured at the University of Buffalo, Monday, March 28, at 4 p. m., under the auspices of the laryngological section of the Buffalo Academy of Medicine. Subject—Diseases of the accessory sinuses with special reference to the maxillary antrum. In the evening Dr. Richards was entertained by the Roswell Park Medical Club.

The Physicians' Club at its annual meeting held March 9, 1904, elected the following-named officers for the ensuing year: president, J. Henry Dowd; vice-president, F. M. Boyle; secretary, W. C. Callanan. Papers were read by Drs. Dowd, J. W. Nash and J. J. Finerty.

The tenth quadrennial congress of Polish Physicians and Scientists, appointed to be held at Lemberg, July 20-24, 1904, has been postponed to an indefinite period on account of the Russo-Japanese war.

